# Computational insights into CRISP3 downregulation in cervical cancer and its cervical lineages pattern

**DOI:** 10.1093/pcmedi/pbae016

**Published:** 2024-07-24

**Authors:** Ricardo Cesar Cintra, Andrés Galindo Céspedes, Mércia Patrícia Ferreira Conceição, Maiza Vitoria Aguiar Silva Oliveira, Alessandro Buron, Deisiane Rodrigues das Neves, Fabio Alves Moraes, Olinda Maria Gamarra, Daniel Rodrigues de Bastos

**Affiliations:** Department of Oncology, Universidade de São Paulo, São Paulo 71.961-540, Brazil; Clinical Pathology Service, Almanzor Aguinaga Asenjo National Hospital, Chiclayo 14001, Peru; Department of Oncology, Universidade de São Paulo, São Paulo 71.961-540, Brazil; Universidad Privada del Este, Alto Paraná 7000, Paraguay; Universidad Privada del Este, Alto Paraná 7000, Paraguay; Universidad Privada del Este, Alto Paraná 7000, Paraguay; Universidad Privada del Este, Alto Paraná 7000, Paraguay; Universidad Privada del Este, Alto Paraná 7000, Paraguay; Universidad Privada del Este, Alto Paraná 7000, Paraguay

**Keywords:** cervical cancer, uterine cervical carcinoma, CRISP3, biomarkers in cancer, prognosis

## Abstract

**Background:**

Cysteine-rich secretory protein 3 (CRISP3) emerges as a potential biomarker in the study of many cancers, including cervical cancer (CC). This study aimed to analyze the expression pattern of CRISP3 in CC patients and CC cell lineages, following treatment with the epigenetic drugs: trichostatin A (TSA) and 5-aza-2'-deoxycytidine (5-aza).

**Methods:**

The differentially expressed genes identified in GSE63514 were used to construct a protein–protein interaction network. CRISP3 was selected for subsequent analyses. We utilized data from the TCGA and GENT2 projects to evaluate the expression profile and clinical behavior of CRISP3. Additionally, we conducted cell culture experiments to analyze the expression profile of CRISP3 in cells.

**Results:**

Low levels of CRISP3 were observed in squamous cell carcinoma (SCC) and human papillomavirus (HPV)16+, along with being associated with worse overall survival (OS). MIR-1229–3p was analyzed, and its high expression was associated with worse prognostic outcomes. In CC-derived cell lines, we observed low levels of CRISP3 in SiHa, followed by SW756, C33A, HeLa, and higher levels in CaSki. All cells were treated with TSA, 5-aza, or both. In all cell lines, treatment with TSA resulted in increased transcription of CRISP3.

**Conclusion:**

We identified a significant downregulation of CRISP3 in CC, particularly in cases with HPV16 infection and SCC, which was associated with poorer OS. Preliminary findings suggest that epigenetic treatments with TSA and 5-aza may modulate CRISP3 expression, warranting further research to elucidate its regulatory mechanisms and potential as a prognostic biomarker.

## Graphical Abstract

**Graphical Abstract:**
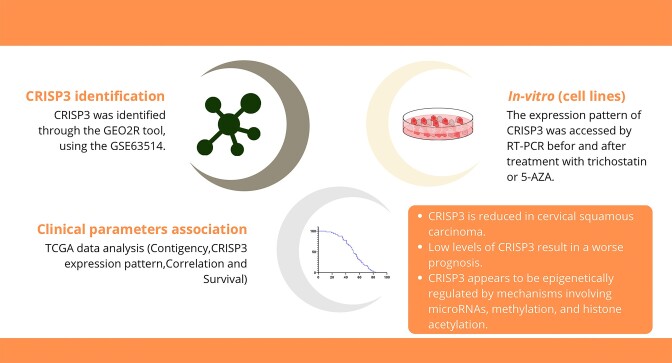

## Introduction

Cervical cancer (CC) is one of the most lethal gynecological neoplasms and the fourth most prevalent cause of morbidity among women worldwide. According to an analysis conducted by the Global Cancer Observatory (GCO), in 2020, there were 604 127 new cases and 341 831 deaths reported due to CC, with most of these numbers coming from low- and middle-income countries [1]. Furthermore, 99.7% of cervical carcinomas are due to high-risk human papillomavirus infection and persistence, considered the primary etiological agent of CC [2].

Despite advances in preventive measures such as human papillomavirus (HPV) vaccination, the variable global coverage, and the lack of efficacy for those already diagnosed or in advanced stages of the disease underscore the need for continued research into novel therapeutic targets. In this context, the cysteine-rich secretory protein 3 (CRISP3) emerges as a potential biomarker. Initially identified as the 28 kDa specific granule protein (SGP28), CRISP3 was first isolated in 1996 from exocytosed material of human neutrophils, marking a significant discovery in the study of secretory proteins [3, 4]. Situated on chromosome 6p12.3, the CRISP3 gene encodes an extracellular matrix protein consisting of 245 amino acids. Physiologically, CRISP3 is found in abundance in plasma, sweat, seminal fluid, and exocrine glands such as the pancreas, salivary glands, and prostate. Conversely, lower levels of CRISP3 are observed in the epididymis, testicles, colon, ovary, lacrimal glands, and thymus [5, 6]. CRISP3 is stored intracellularly in eosinophil and neutrophil granules, in glycosylated or non-glycosylated form, and is secreted for innate host defense, succeeding in the degradation of the extracellular matrix through proteolytic enzymes [5, 3]. Although the exact function of CRISP3 remains unknown, its structural similarity to proteins involved in pathogenic processes, along with its occurrence in exocrine secretions of epithelial tissues, suggests its involvement in immune responses and inflammatory processes, as well as indicating a potential role in the pathophysiology of various diseases [6, 7].

In the prostate, CRISP3 levels are reduced; however, in cancerous contexts of this tissue, overexpression is observed, with significantly increased levels ranging from 20 to 2 000 times more [8]. In the context of prostate cancer, the CRISP3 promoter is regulated by the androgen receptor (AR) through an epigenetic mechanism, and CRISP3 expression is induced by the presence of dihydrotestosterone (DHT) in AR-positive cells. This is evidenced by CRISP3 promoter activity, which was reduced in cells cultured in steroid-free medium and was restored after treatment with DHT [9]. In lung cancer, high levels of CRISP3 are observed, and even higher levels are seen after chemotherapy treatment, correlating with a metastatic profile [10].

On the other hand, a study demonstrated CRISP3 in an axis with miR-508–5p and LINC01342, where both CRISP3 and LINC01342 silencing resulted in decreased cell growth capacity, colony formation, invasion, and metastasis [11]. In breast neoplasms, CRISP3 overexpression is associated with tumor spread and severity [12]. Similarly, in esophageal cancer, high levels of CRISP3 have been associated with cell proliferation and metastases, leading to poor prognosis [7]. Immunostaining in ovarian epithelium, benign tumors, and ovarian cancer (OC) specimens did not show variations for CRISP3 protein distribution [13]. Conversely, another study found that transcriptional reduction of CRISP3 is an independent prognostic factor and is associated with lower overall survival (OS) of OC patients [14]. In oral squamous cell carcinoma, a decrease in CRISP3 expression was observed, especially in early stages, where loss of CRISP3 DNA copy number was detected [15]. In the context of CC, although only one study addressed CRISP3, there was no specific attention directed to this gene. In this study, normal tissues, low- and high-grade intraepithelial lesions, as well as squamous cell carcinoma were examined through immunohistochemistry. Notably, only normal tissues showed positive staining for CRISP3 [16].

To enhance our understanding of the relationship between CRISP3 and CC, acknowledging that there is no fixed pattern suggesting a consistent role for CRISP3 in the tumorigenic process and that it appears to vary depending on the tumor type and stage of tumorigenesis, we evaluated CRISP3 expression in CC tissues and examined its prognostic significance using public databases. We extended our investigation by assessing CRISP3 expression in representative cervical tumor-derived cell lines.

## Material and methods

### Selection of datasets and analysis of differential genes expression

**Figure 1.**
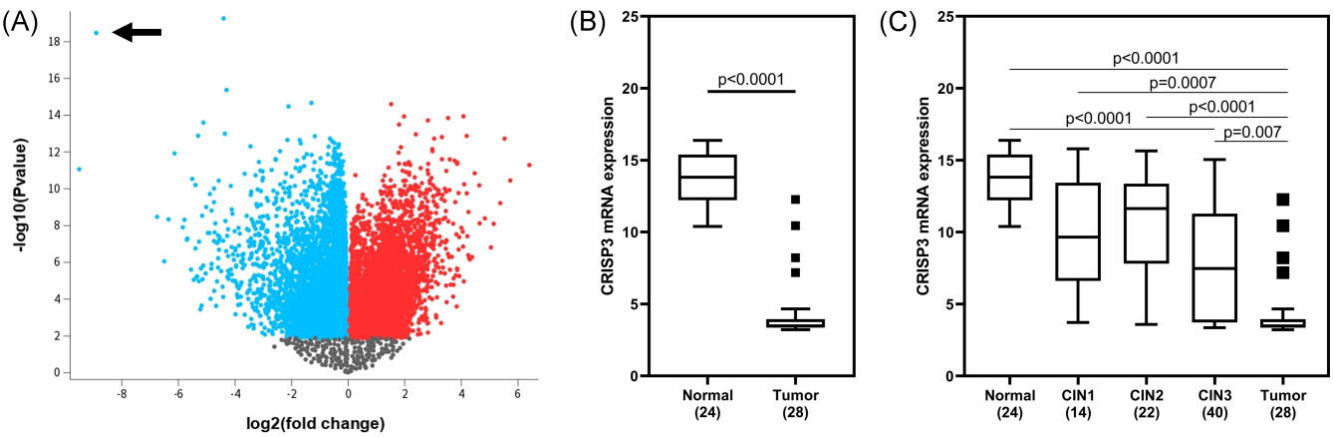
CRISP3 identification and expression profile. (**A**) Volcano plot containing the 54 675 probes analyzed in GSE63514 from the identification group. Blue corresponds to probes with fold-change value < 0 and p.adj value < 0.05 in tumor tissue. Red corresponds to probes with fold-change value > 0 and p.adj value < 0.05 in tumor tissue. The region with the highest number of overlapping points, in black, corresponds to probes with a *P*-value without statistical significance. The black arrow indicates the CRISP3 probe. (**B**) CRISP3 expression profile in normal tissue and tumor tissue samples from the identification group. (**C**) CRISP3 expression profile in normal tissue, grade 1, 2 and 3 of cervical intraepithelial neoplasia (CIN), and tumor tissue. The data were tested for normality and subjected to the Mann–Whitney and Kruskal–Wallis U Test with Dunn's multiple comparison test.

The gene expression datasets under analysis were sourced from the Gene Expression Omnibus database (https://www.ncbi.nlm.nih.gov/geo/). Specifically focusing on datasets associated with CC, an exhaustive search was conducted within the database, and after meticulous screening, we identified the microarray dataset GSE63514 [17]. Employing GEO2R (www.ncbi.nlm.nih.gov/geo/geo2r), we screened and isolated differentially expressed genes (DEGs) based on a significance threshold, where Benjamini–Hochberg < 0.05, and |logFC|≥1.5 (normal vs. tumor). A box plot (supplementary Fig. S1, see online supplementary material) was constructed with GEO2R to standardize and assess the dataset samples. Additionally, to visually represent the DEGs, a volcano plot was generated (Fig. 1A), providing a graphical overview of the gene expression alterations. From this analysis, the probe that displayed the lowest adjusted *P*-value (p.adj) was singled out for an in-depth investigation into its associations with the clinicopathological features of patients diagnosed with CC, utilizing data from The Cancer Genome Atlas (TCGA) study.

### Function and pathway enrichment analysis and protein–protein interaction network

The top 100 DEGs were used in the STRING database (https://www.string-db.org/) to generate the protein–protein interaction (PPI) network and enrichment pathway [18]. A confidence interaction score of 0.4 was employed as the significance threshold. Subsequently, the PPI network was visualized utilizing Cytoscape software v3.10.1 (www.cytoscape.org/) [19].

### TCGA and GENT2 data analysis

The cBioPortal platform (https://www.cbioportal.org/) provided access to the Firehose Legacy TCGA public data, from which the mRNA CRISP3 expression profiles of 310 patients were acquired. Among these, 4 cases lacked expression information and were consequently excluded from the study, resulting in a cohort of 306 patients eligible for evaluation. After this exclusion, an integration of the expression data with clinical-pathological information was conducted to investigate the correlation between CRISP3 gene expression and various clinical and pathological parameters. Median gene expression values were computed to stratify the patient groups into low (≤median) and high (>median). Following this classification, a contingency table was constructed, and statistical analysis was performed employing either the chi-squared test or Fisher's exact test, as appropriate. The survival analysis utilized the Kaplan–Meier (KM) method and log-rank test, employing the same 'high' and 'low' stratification. For a comprehensive quantitative examination of CRISP3 expression profiles, an initial assessment was made to ascertain the Gaussian distribution of the data. Subsequently, depending on the data distribution characteristics, statistical analyses such as the t-test or Mann–Whitney test were applied for paired variables, while analysis of variance (ANOVA) or Kruskal–Wallis tests were employed for comparisons among three or more groups. The presentation of CRISP3 expression involved log-transformed mRNA expression z-scores, which were juxtaposed with the overall expression distribution of all samples (RNA Seq V2 RSEM). Additionally, the beta value (HM450) was utilized to indicate the DNA methylation levels of individual CpG sites in comparison to the corresponding mRNA expression, as described in previous studies [20, 21]. GENT2 provides differential expression analysis and prognosis assessment based on normal and tumor tissues, including cell lineages. All data in GENT2 is sourced from TCGA and GEO databases [22]. The keyword CRISP3 was utilized to access mRNA expression data in tissues and CC cells. Expression data files for GPL570 platform (HG-U133_plus_2) and GPL96 platform (HG-U133A) were acquired, and statistical analysis was performed. To evaluate the difference between normal and tumor tissue, the Mann–Whitney test was employed.

### microRNA prediction, and survival association

Detection and analysis of microRNAs (miRNAs) that target CRISP3 was performed using the mirTarBase database, (mirtarbase.mbc.nctu.edu.tw) which contains miRNAs experimentally validated. In addition, miRWalk (zmf.umm.uni-heidelberg.de/), was also employed to predict miRNAs by analyzing statistically significant interactions (*P* < 0.05) [23, 24]. The prognostic value of selected miRNAs was determined using the TCGA database (Firehose Legacy study). All microRNAs were evaluated for overall survival (OS) and relapse-free survival (RFS) across all subtypes, including cervix squamous cell carcinomas. For statistical analysis, the KM method with log-rank test comparison was applied by selecting the optimal cutoff from the CC TCGA cohort.

### Cell culture and 5-aza-2'-deoxycytidine and trichostatin A treatment

The following cervical cancer-derived cell lines were utilized: C33A (HPV-negative; ATCC^®^ CRM-HTB-31™), SiHa (HPV16; ATCC^®^ HTB-35™), SW756 (HPV18; ATCC® CRL-10302TM), HeLa (HPV18; ATCC^®^ CCL-2™), and CaSki (HPV16; ATCC^®^ CRL-1550™); all cell lines were cultured in Minimum Essential Medium (MEM) (GibcoTM, Invitrogen, USA) with 10% fetal bovine serum (FBS) (Gibco). Authentication and mycoplasma testing were conducted for all cell lines used. Maintenance was carried out at 37°C and 5% CO_2_ (Thermo Electron Corporation, USA). Prior to the commencement of the expanded experiment across all cell lines, preliminary toxicity assays were conducted for trichostatin A (TSA) and 5-aza-2'-deoxycytidine (5-AZA) to determine non-toxic drug concentrations for use in subsequent treatments. Based on these assays, uniform drug concentrations were selected for the treatment of SiHa, HeLa, SW756, and CaSki cell lines. Treatments were administered either as single-agent or in combination. However, for the C33A cell line, which also received the same treatments, the TSA dose was halved in the combined treatment regimen to mitigate toxicity. The treatment dosages were as follows: for SiHa, HeLa, SW756, and CaSki cell lines, the TSA concentration was set at 75 nM, 5-AZA at 10 µM, and for the combined treatment of 5-AZA with TSA, the concentration was 7.5 µM of 5-AZA plus 75 nM TSA. In contrast, the C33A cell line was treated with a reduced TSA concentration of 37.5 nM, with 5-AZA remaining at 10 µM, and for the combined treatment, the concentration was 7.5 µM of 5-AZA plus 37.5 nM TSA. Due to the compound's short half-life in culture media, the 5-AZA medium was prepared and replaced daily for 7 days.

### RNA isolation and reverse transcription-polymerase chain reaction

RNAs extracted from the cell cultures were isolated using TRIzol reagent (Invitrogen), following the manufacturer's instructions. The RNA samples underwent treatment with DNAase RQ1 (Promega Corp., USA). cDNA synthesis was performed using the Go ScriptTM Reverse Transcription System Kit (Promega Corp.). Quantitative reverse transcription polymerase chain reaction (RTq-PCR) were conducted using Go Taq® qPCR Master Mix (Promega Corp.). Primers specific for CRISP3 and the housekeeping gene glyceraldehyde-3-phosphate dehydrogenase (GAPDH) were synthesized by Thermo Fisher Scientific (USA). The primer sequences (from 5′ to 3′) were as follows: human CRISP3 forward: TGCTCTGGAAACCACTGCAA, human CRISP3 reverse: CAGCAACCAGGAACAACAGC, and human GAPDH forward: GACTGTGGTCATGAGTCCTCCC, human GAPDH reverse: CAAGATCATCAGCAATGCCTCC. The RT-qPCR reactions were processed using an ABI Prism 7500 instrument (Applied Biosystems, USA), and the delta-delta CT method was applied for quantification.

### Statistics

For statistical analysis, SPSS (Statistical Package for Social Sciences) version 25.0 (IBM Inc., Armonk, NY USA) or GraphPad v.7 (California, USA) was used. The chi-square or Fisher's exact test was applied to compare categorical variables. A Gaussian distribution test was performed on all analyzed groups. The Mann–Whitney or t-test was used to evaluate the difference between two groups, and ANOVA or Kruskal–Wallis for evaluation in more than two groups. Correlation analysis was carried out by Spearman test with a 95% confidence interval (CI). For survival analysis, the curves were performed using the KM method with comparison using the log-rank test; in addition, Cox regression was performed, and the result was demonstrated as a hazard ratio (HR) with a 95% CI. A significance level of 5% was adopted.

## Results

In the identification group, from study GSE63514, a total of 54 675 probes were examined, corresponding to individual genes, of which 13 908 showed an adjusted *P*-value (p.adj) < 0.05 (Fig. 1A). Of these, probe 207 802_at stands out for having the lowest p.adj value on a logarithmic scale and corresponding to the CRISP3 gene. In the same population, CRISP3 was isolated and graphically depicted, showing its significant reduction in cervical tumors (Fig. 1B). In addition, samples of cervical intraepithelial neoplasia (CIN) grades 1 to 3 were included. An apparently progressive decrease in CRISP3 expression appears to occur according to the severity of the lesion, although in comparison with normal tissue it is significant only for CIN3 and tumors (Fig. 1C).

**Figure 2.**
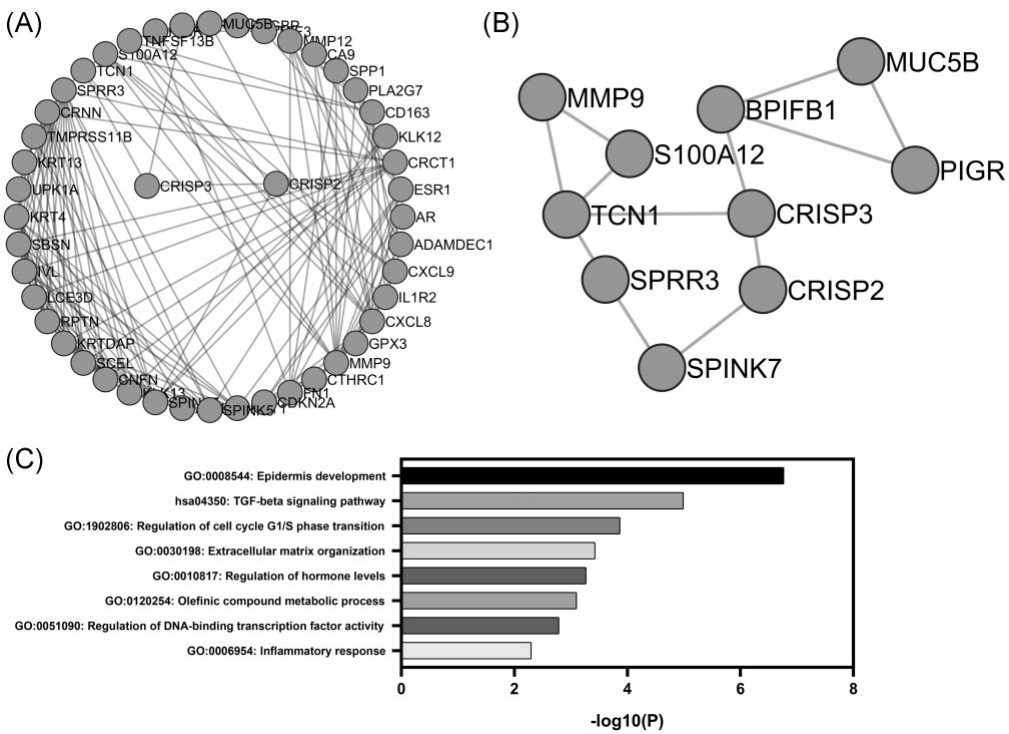
PPI network and pathway enrichment analysis. (**A**) The PPI network was constructed using the STRING database, employing the top 100 deregulated genes identified in the GSE63514 dataset. (**B**) A cropped and enlarged version of (A), focusing on genes closely associated with CRISP3. (**C**) The enrichment analysis prominently highlights the main altered pathways, providing an in-depth insight into the functional implications arising from protein interactions.

With the objective to investigate the functions related to the deregulation of CRISP3 in the context of uterine colon cancer, we performed a protein–protein interaction analysis using the String database and Cytoscape for network visualization. Among the DEGs co-expressed with CRISP3 are classic markers such as the estrogen receptor (ESR1) and the AR, as well as interleukins and interleukin receptors (Fig. 2A). Next, we highlight the closest interactions with CRISP3: where CRISP2 is observed, which shares affinities as a member of the same protein family; transcobalamin 1 (TCN1), a member of a family of vitamin B12-binding proteins associated with immune infiltration [25]; and a BPI plug that contains member 1 of family B (BPIFB1), known for its involvement in innate immune response to bacterial exposure (Fig. 2B) [26]. In addition to this, we performed a pathway enrichment analysis, from which we could observe a significant role of these genes in 'epidermal development', 'TGF-β signaling pathway', 'cell cycle regulation', 'extracellular matrix organization', 'regulation of hormonal levels', 'olefinic compound metabolic process', 'regulation of DNA-binding transcription factor activity' and 'inflammatory response' (Fig. 2C).

After identifying and understanding CRISP3 more profoundly, we aimed to look for possible associations with the clinicopathological characteristics of patients with uterine CC. To do this, we accessed the TCGA project's RNA-seq dataset through the cbioPortal. The median expression of CRISP3 levels of all samples was calculated. Patients were then grouped into high (above the median) or low (below the median). We observed significant associations between CRISP3 and patient age (*P* = 0.0098), in terms of histological subtype (*P* = 0.0022), and in terms of HPV type (*P* = 0.0001). No associations were observed between CRISP3 and history of contraceptive use, menopausal status, tumor staging, or histological grade (Table 1).

**Table 1.** Clinicopathological features based on CRISP3 expression in patients with CC from the TCGA—Firehose Legacy study.

Parameters	Low expression		High expression		
	*n*	%	*n*	%	*P*-value
**Age**					
≤50	83	54.2	105	68.6	0.0098
>50	70	45.8	48	31.4	
**History of hormonal contraceptives use**					
Current user	5	6.0	10	13.3	0.1442
Former user	26	31.3	28	37.3	
Never used	52	62.7	37	49.4	
**Menopause status**					
Peri	8	6.5	17	15.5	0.0513
Pre	66	53.7	60	54.5	
Post	49	39.8	33	30.0	
**Cancer type detailed**					
SCC	138	90.2	115	75.2	0.0022
Adeno	7	4.6	20	13.0	
Others	8	5.2	18	11.8	
**HPV status**					
HPV16	52	59.1	51	56.0	0.0001
HPV18	3	3.4	24	26.4	
Others	26	29.5	13	14.3	
Negative	7	8.0	3	3.3	
**Stage (TNM)**					
I/II	112	75.7	119	78.8	0.5183
III/IV	36	24.3	32	21.2	
**Neoplasm histologic grade**					
1	7	4.9	12	9.2	0.3926
2	70	48.9	65	49.6	
3	65	45.5	54	41.2	
4	1	0.7	0	0.0	

The low and high classification was accessed by mRNA expression z-score median value. SCC: squamous cervical carcinomas. Adeno: adenocarcinomas.

**Figure 3.**
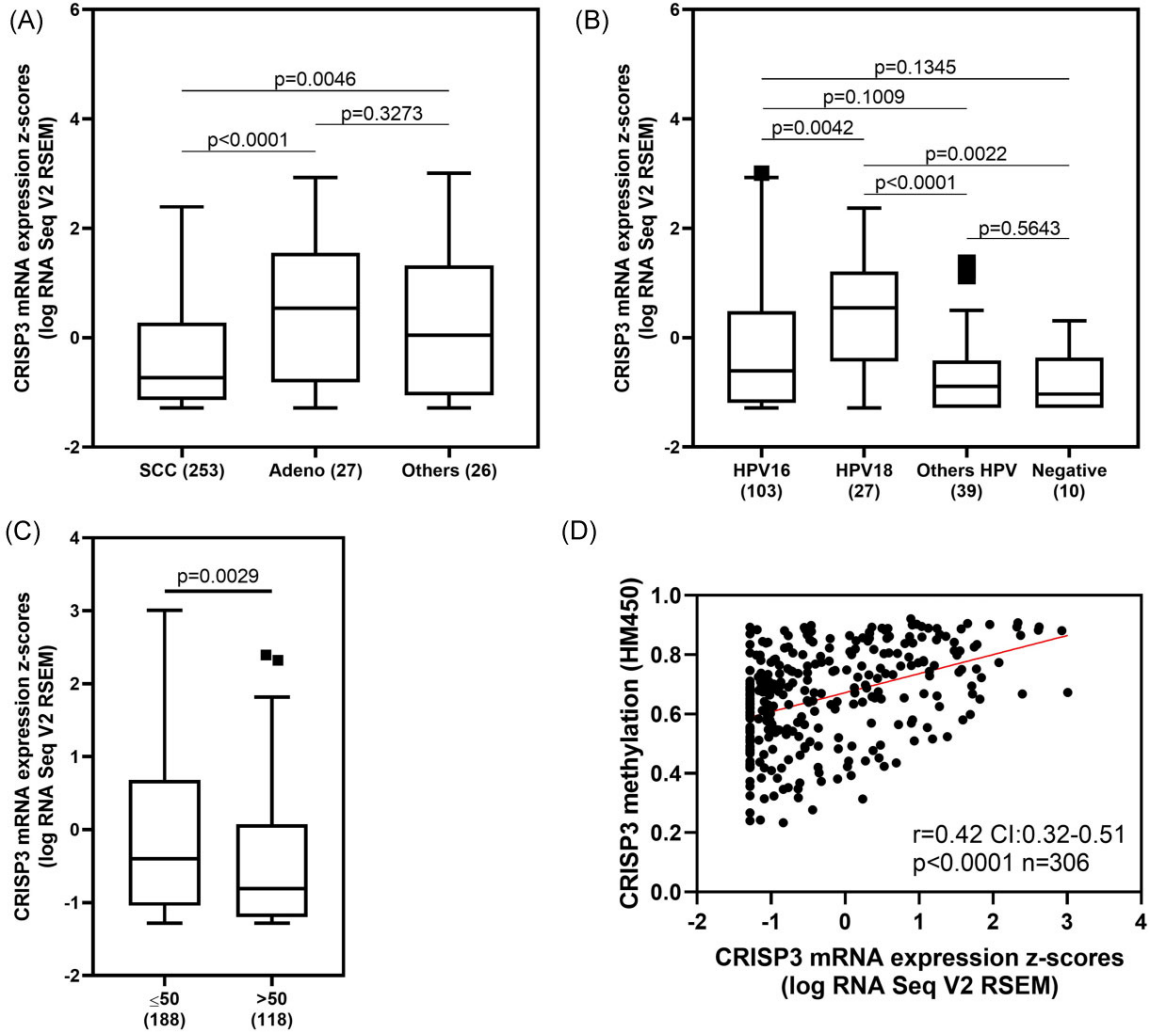
CRISP3 RNA levels in CC by (**A**) histological subtype categorized in SCC, Adeno, and other subtypes; (**B**) HPV type. Kruskal–Wallis test was applied with Dunn's multiple comparison test; and (**C**) age. Mann–Whitney test was applied. (**D**) Correlation between CRISP3 RNA levels and methylation was accessed using the Spearman test. The analyses are based on TCGA Firehose Legacy CC patients. SCC: squamous cervical carcinomas. Adeno: adenocarcinomas.

Still in relation to this same population from the TCGA study, we evaluated possible differences in CRISP3 transcriptional levels depending on clinical pathological data. CRISP3 levels were significantly lower in patients with squamous cell carcinoma (SCC), compared with adenocarcinoma (*P* < 0.0001; Fig. 3A) and other histological subtypes (*P* = 0.0046; Fig. 3A). Due to the characteristics of the E6 and E7 oncoproteins produced by different types of HPV, we also analyzed whether there could be differences in CRISP3 transcriptional levels. We observed that tumor samples positive for HPV18 have a significantly higher mean expression of CRISP3 than other viral types (*P* < 0.05; Fig. 3B). As already demonstrated in the association analysis in Table 1, we also investigated possible differences depending on age and observed that patients > 50 years of age present a reduction in CRISP3 levels. Furthermore, we observed a positive correlation between CRISP3 methylation and its transcriptional levels (*P* < 0.0001; Fig. 3D).

**Figure 4.**
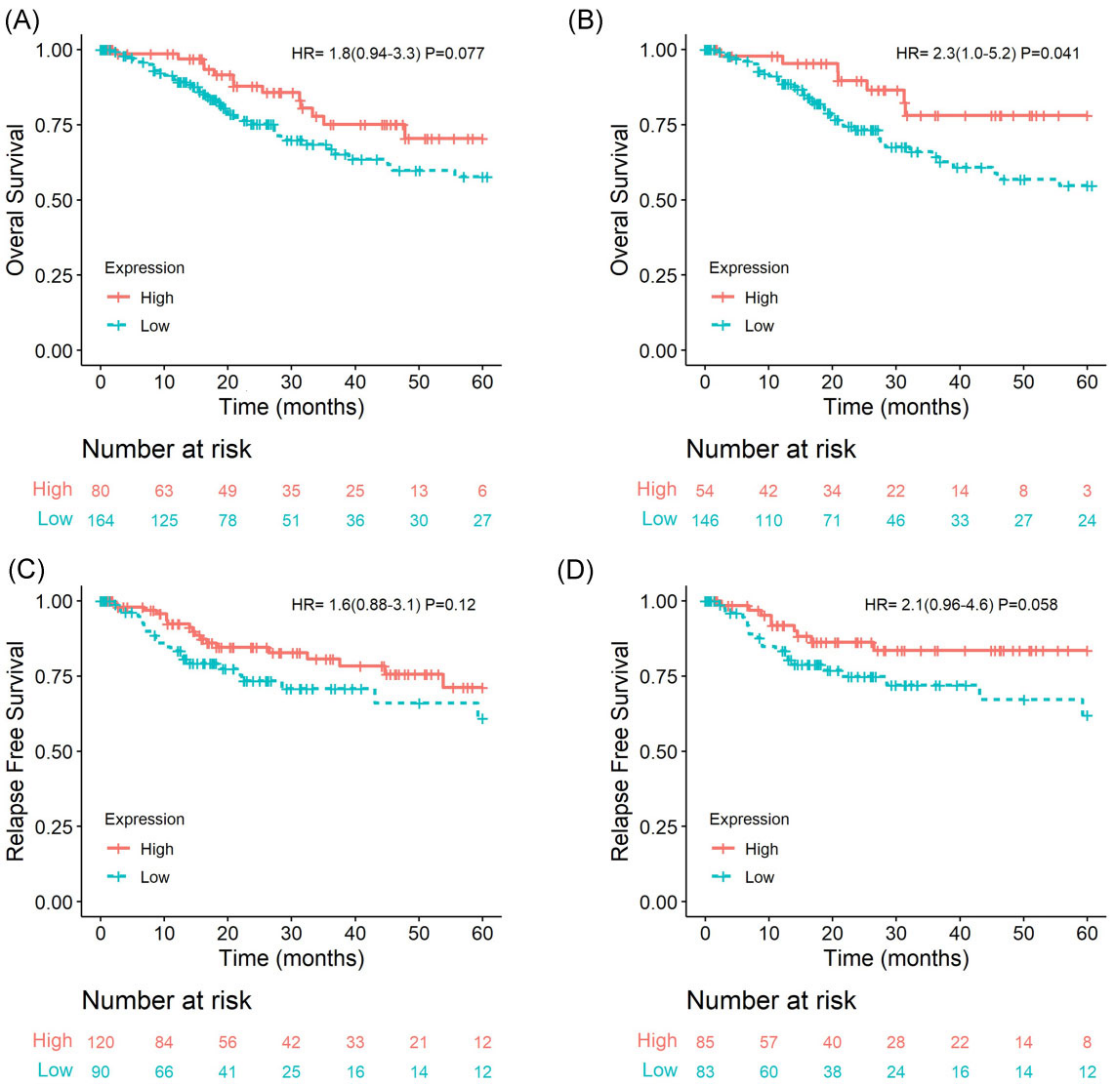
OS of patients with uterine CC with CRISP3 expression (**A**) regardless of histological subtype and (**B**) SCCs of the uterine cervix subtype. RFS of patients with uterine CC with CRISP3 expression (**C**) regardless of histological subtype and (**D**) only SCCs of the uterine cervix. Patients were categorized according to the best cutoff of CRISP3 mRNA expression. Data from the TCGA Firehose study.

Regarding the prognostic role of CRISP3, we did not observe an association with OS in all CC patients (HR = 1.80, 95% CI: 0.94–3.30; *P* = 0.077; Fig. 4A) nor with RFS in all CC patients (HR = 1.60, 95% CI: 0.88–3.10; *P* = 0.12; Fig. 4C). Stratifying the samples only by the SCC histological subtype, we observed that low CRISP3 expression is associated with a worse prognosis in OS (HR = 2.30, 95% CI: 1.00–5.20; *P* = 0.041; Fig. 4B), but not in RFS (HR = 2.10, 95% CI: 0.96–4.60; *P* = 0.058; Fig. 4D).

**Figure 5.**
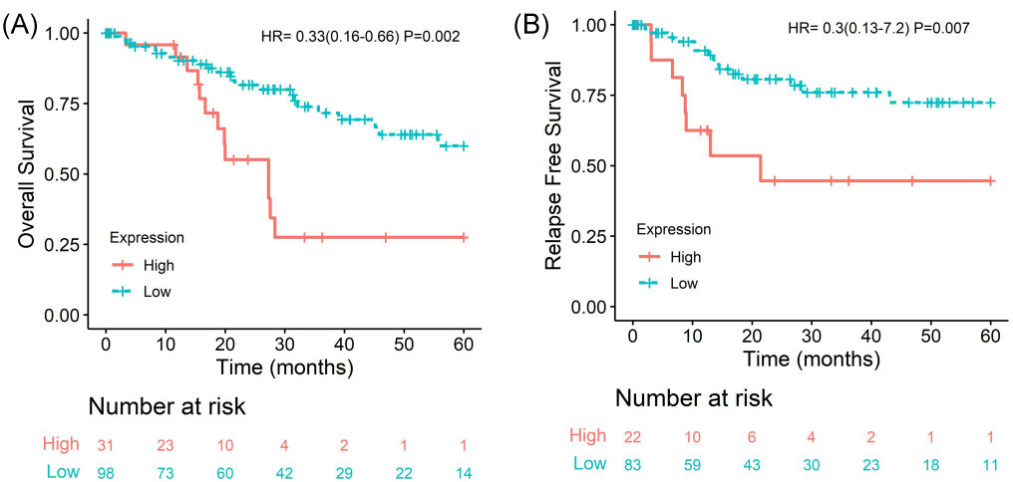
Survival analysis of patients with SCCs of the uterine cervix stratified by has-miR-1229–3p expression. (**A**) OS and (**B**) RFS. Patients were categorized according to the best cutoff of miRNA expression. Data from the TCGA Firehose study.

We analyzed miRNAs capable of interacting with CRISP3 mRNA. By using miRTarBase, we identified hsa-miR-1229–3p as a potential regulator of CRISP3. In the survival analysis, elevated levels of hsa-miR-1229–3p showed a significant association with worse OS (HR = 0.33, 95% CI: 0.16–0.66; *P* = 0.002; Fig. 5A) and poor RFS (HR = 0.30, 95% CI: 0.13–0.72; *P* = 0.007; Fig. 5B). By using miRWalk 18, 3′ untranslated regions (3′ UTRs) and 13 CDS targeting sequences were identified. Among these, only hsa-miR-508–5p and hsa-miR-3614–5p were evaluated for OS and RFS. Elevated levels of hsa-miR-3614–5p were associated with worse OS (HR = 0.43, 95% CI: 0.18–1.00; *P* = 0.049; supplementary Fig. S2C, see online supplementary material). No significant association was found for hsa-miR-508–5p (supplementary Fig. S2).

**Figure 6.**
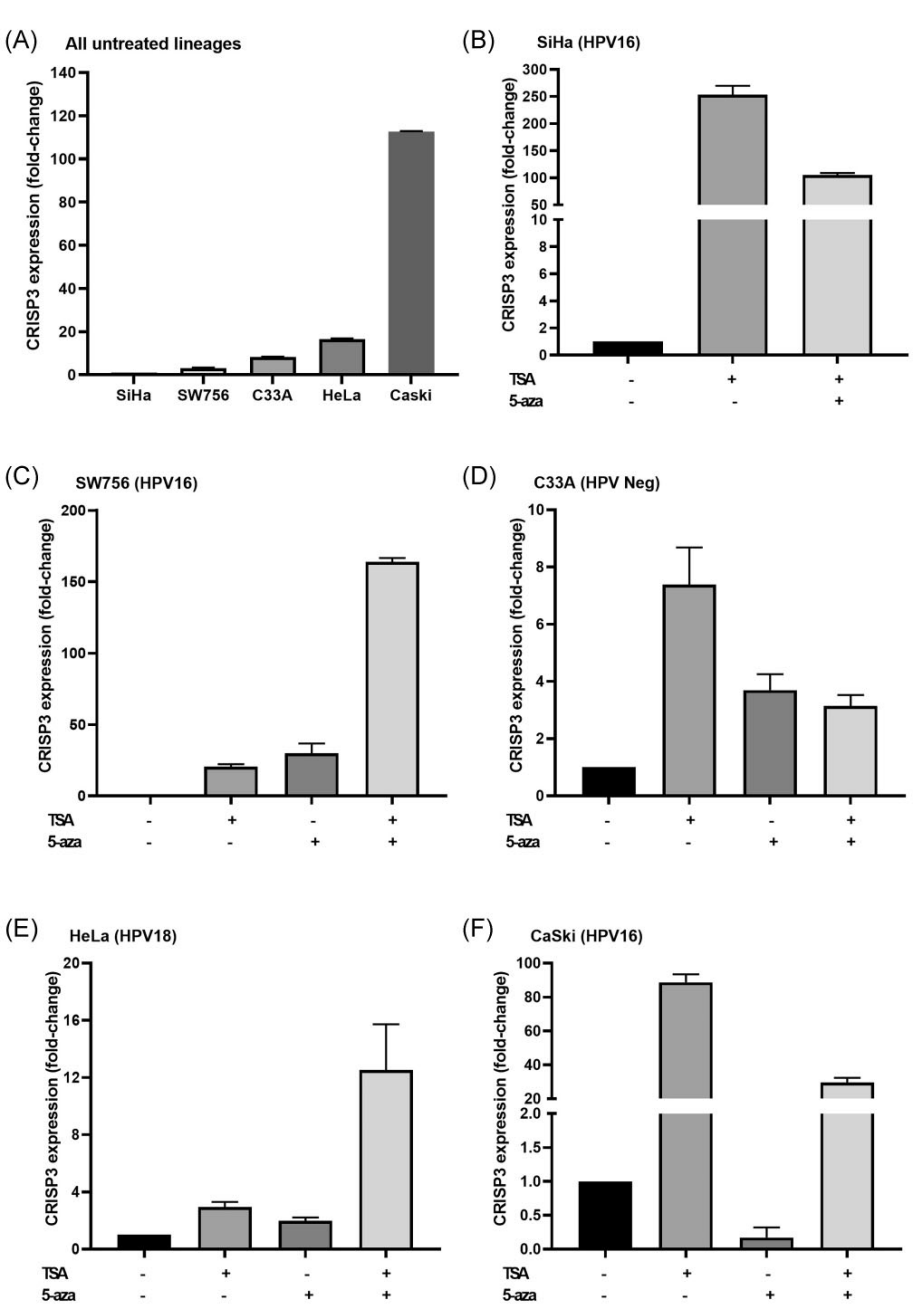
(**A**) CRISP3 expression in CC cell lineages. CRISP3 expression under TSA or 5-AZA treatment in: (**B**) SiHa, (**C**) SW756, (**D**) C33A, (**E**) HeLa, and (**F**) CaSki. All RT-PCR experiments were conducted in triplicate.

Finally, we analyzed the transcriptional pattern of CRISP3 in cell lines derived from CC. We observed that SiHa has a reduced expression of CRISP3, while SW756 exhibits a fold-change of 3.1. Both C33A and HeLa show a CRISP3 expression 8.3 and 16.5 times higher than SiHa, respectively, while for CaSki, this increase can be up to 112.6 times higher (Fig. 6A). Treatment with TSA effectively increased the transcriptional levels of CRISP3 in all analyzed cell lines (Fig. 6B–F). Similarly, treatment with 5-AZA also led to an increase in CRISP3 expression, although a transcriptional reduction was observed in CaSki (Fig. 6F). The combined treatment of TSA and 5-AZA resulted in enhanced CRISP3 expression across all cell lines, with an additive effect noted in SW756 cells (Fig. 6C) and HeLa cells (Fig. 6E).

## Discussion

The quest for novel cancer biomarkers is a matter of global importance, with substantial progress arising from bioinformatics. While CC can be effectively prevented through screening and HPV vaccination, the continued incidence of new cases is influenced by variable vaccination coverage, the impact of anti-vaccine movements, and regional disparities in the adoption of preventive practices [27, 28]. It is critical to acknowledge that while vaccination targets the viral types commonly linked to CC, it does not eradicate the possibility of infection with other high-risk HPV types. Moreover, social behavior might facilitate the spread of less frequent types not covered by the vaccine. In this regard, understanding the patterns and mechanisms by which specific genes drive cancer development is crucial for elucidating oncogenic processes and promoting the development of new targeted therapies. Additionally, there is a significant number of women around the world living with CC who cannot benefit from vaccination, requiring special attention from the scientific community. In this sense we conducted this work with the aim of identifying new biomarkers that may serve as prognostic support in management and therapeutics of the disease.

Through a computational approach, we identified the differential expression of CRISP3, characterized by significantly reduced levels in cervical tumors in comparison with normal tissue. Additionally, we conducted an analysis using the GENT2 platform and observed that, in other populations, the expression of CRISP3 is also significantly reduced in tumor samples (supplementary Fig. S1). These findings may be slightly supported by the study conducted by Li *et al*. [16]. In this study, Li *et al*. selected DEGs for an IHC screening, which included CRISP2, CRISP3, Ki67, CDKN2A, KRT17, SYCP2, NEFH, DSG1, and PTG in samples of normal tissue, low-grade lesions (LSIL), high-grade lesions (HSIL), and SCC of the uterine cervix [16]. During this screening, CRISP3 was not selected based on the authors' criteria and, therefore, was not further explored. However, the absence of CRISP3 staining in LSIL, HSIL, and SCC, in contrast to its positivity in normal tissue samples, draws our attention. Our study differs from Li *et al*.'s work and other reports by using a comprehensive bioinformatics approach to analyze gene expression data from a large TCGA dataset. While other studies may not have reported CRISP3 expression due to methodological differences and sample types, our detailed analysis reveals specific patterns of CRISP3 expression across different histological subtypes and in relation to HPV status. Additionally, we provide a transcriptional profile of CRISP3 in different cervical tumor cell lines and its alterations in response to treatment with drugs that regulate epigenetic factors.

In our analysis, we observed that intraepithelial lesions presented a transcriptional reduction of CRISP3 in an apparent progressive decline from normal tissue to high-grade lesions (CIN3) and CC. Confirmation of this hypothesis in the analyzed patient group was challenging, partly due to the small sample size. Nevertheless, the medians of CIN1 and CIN2 are relatively lower than those of normal tissue, while the disparity between normal tissue and CIN3 is statistically significant. This pattern suggests a potential association between reduced expression of CRISP3 and the pathological progression of the uterine cervix, indicating potential biomolecular implications in cervical carcinogenesis. Furthermore, CRISP2 emerges as one of the key DEGs with a logFC of −5.115 and p.adj of 1.26e-10 (supplementary Table S1, see online supplementary material) identified in our study. After statistical analysis, we observed a high positive correlation with CRISP3 (*r* = 0.86; 95% CI: 0.80–0.90; *P* < 0.0001; supplementary Fig. S3, see online supplementary material). Based on this and the proximity of these genes as members of the same family, we analyzed the CRISP2 profile, and a similar pattern to CRISP3 was observed, likely indicating a progressive decrease (supplementary Fig. S3B). Supporting these findings, in Li *et al*.'s work, the staining for CRISP2 was significantly lower in HSIL and SCC, with high medians in normal tissue and LSIL [16].

During gestation, a recurring observation is the decrease in CRISP3 expression, an event that correlates with the elevation of human chorionic gonadotropin (hCG) hormone levels. Scientific literature suggests that this reduction in CRISP3 expression may be associated with temporary and selective suppression of immune function at the implantation site, thereby facilitating blastocyst nesting and subsequent establishment of pregnancy [29]. This phenomenon is of particular interest when considering the documented association between multiparity and increased susceptibility to cervical carcinoma [30]. The conjecture arising from this observation is that decreased levels of CRISP3, induced by hCG elevation during gestation, could contribute to an increased risk of developing cervical oncological pathologies. The proposed explanation for this mechanism is that temporary attenuation of local immune response could favor an environment for progression of pre-existing infections, particularly those of viral etiology, such as HPV infection, already known for its etiological relationship with CC.

Conversely, *in silico* research revealed an increase of CRISP3 expression in patients diagnosed with multiple myeloma (MM). In this study, researchers proceeded with analysis using total RNA extracted from peripheral blood samples, which included 18 healthy controls and 12 MM patient samples. The results obtained through RT-qPCR significantly confirmed the CRISP3 overexpression in MM [31]. Thus, differential expression of CRISP3 seems to emerge as a potential biomarker not only in established tumors but also in other human malignancies, reinforcing the idea of studying CRISP3 more comprehensively. Similarly, we not only identified CRISP3 as a differentially expressed gene but also as a potential prognostic biomarker. Initially, we assessed its prognostic role independent of histological subtype. However, only after stratifying the study population did we identified a significant association between low levels of CRISP3 and worst OS in patients with SCC.

Additionally, we conducted an analysis to identify miRNAs with the potential to regulate CRISP3. miR-1229–3p was the only one retrieved from the miRTarBase public database and was significant associated with worse OS and RFS. In addition, we identified the miRNAs miR-508–5p and miR-3614–5p. According to the literature, high levels of miR-508–5p are observed in patients with lung cancer. In the same study, the authors demonstrate that high levels of CRISP3 and a long non-coding RNA (lncRNA), LINC01342, suggesting that the increase in CRISP3 in these tumors is due to the competitive effect promoted by the lncRNA [11]. In the context of CC, we hypothesize that highly complex epigenetic regulation may occur, including post-transcriptional regulation mediated by miRNAs. Additionally, factors such as DNA methylation and histone acetylation seem to contribute to the downregulation of CRISP3, which led to the motivation for subsequent analyses.

In our *in vitro* analysis, we observed that HPV16-positive cell lines (SiHa and SW756) exhibit a lower fold-change value, while the HPV-negative C33A and HPV18-positive HeLa cells show fold-change values higher than SiHa by 8.3 and 16.5 times, respectively. Interestingly, in our *in silico* analyses, we found lower transcriptional levels of CRISP3 in patients positive for HPV16. Furthermore, in the *in silico* analysis, we observed significantly decreased levels in patients with SCC, and the SiHa cell line, derived from squamous carcinoma, also showed a reduced transcriptional profile. However, CaSki, also known to be HPV16 positive, exhibited the highest levels of CRISP3. For this cell line, a cautious evaluation is warranted, considering two substantial characteristics that drastically alter its behavior: the multiple copies of integrated HPV16 in its genome (>600 copies) and its derivation from a metastatic SCC. Regarding treatment, TSA was able to increase CRISP3 transcription in all analyzed tumor cell lines, suggesting that epigenetic regulation based on histone activity may be present. Interestingly, in Pathak *et al*.’s study [9], the CRISP3 promoter activity was measured using luciferase activity. In PC3 and RWPE-1 cells, both CRISP3-negative, it was demonstrated that the CRISP3 promoter is silenced by histone deacetylation [9]. Treatment with 5-AZA also led to a transcriptional increase in CRISP3 in the analyzed cell lines, except in CaSki. In our *in silico* analysis, we found a positive correlation between methylation and CRISP3 mRNA, which contradicts the idea of a simple promoter regulation. However, it is worth noting an analysis by Pongor *et al*. [32] that examined global DNA methylation in human small cell lung cancer. Although not focusing on CRISP3, one of their analyses shows a pattern of both hyper- and hypo-methylation in both the promoter and the gene body, despite the reduced levels in these cell lines [32]. Finally, in cell lines such as SW756 and HeLa, an even more sophisticated regulation seems to occur, as the combined treatment of TSA and 5-AZA resulted in higher transcriptional activity of CRISP3. Although we observed that HPV18-positive samples showed significantly higher CRISP3 expression, the comparable expression of CRISP3 in HPV-negative cell lines suggests that other molecular factors may be involved. Future studies should investigate the specific regulatory mechanisms of CRISP3 in relation to different HPV types to clarify this complex relationship. Finally, the regulation of CRISP3 expression may be influenced by various signaling pathways and transcription factors. Previous studies suggest that CRISP3 expression can be modulated by epigenetic factors and specific protein interactions [9]. In our study, we observed that treatment with epigenetic regulators can significantly influence the transcriptional levels of CRISP3. This suggests that the regulation of CRISP3 is tightly controlled by robust epigenetic mechanisms, as well as potential effects of miRNAs. This insight adds a new dimension to our understanding of CRISP3’s role in CC, although additional studies are needed to fully understand the extensive molecular mechanisms regulating this gene.

We acknowledge that our study has limitations, including the lack of additional experimental validation in biological samples and the reliance on gene expression data from public databases. Additionally, the relationship between CRISP3 and different HPV types is not fully elucidated, requiring further studies to confirm our findings.

## Conclusion

In the course of our research, we pinpointed CRISP3 as the predominant gene exhibiting differential expression between normal and neoplastic tissues. Notably, we documented a decline in CRISP3 expression in patients diagnosed with SCC and concurrent HPV16 infection, a phenomenon that was associated with a diminished OS rate. Additionally, our exploration into the modulatory effects of both isolated and synergistic applications of TSA and 5-AZA on CRISP3 expression yielded encouraging outcomes. Despite these advances, further research is imperative to elucidate the intricate regulatory mechanisms governing CRISP3 and to ascertain its potential role in the ongoing surveillance and management of individuals affected by CC.

## Supplementary Material

pbae016_Supplemental_File
