# Supplementary material for: Computational insights into CRISP3 downregulation in cervical cancer and its cervical lineages pattern
Source: Precis Clin Med. 2024 Jul 24;7(3):pbae016. doi: 10.1093/pcmedi/pbae016 (PMC11319933; doi:10.1093/pcmedi/pbae016)
Supplement: pbae016_Supplemental_File [file pbae016_supplemental_file.docx]

**Supplementary materials**


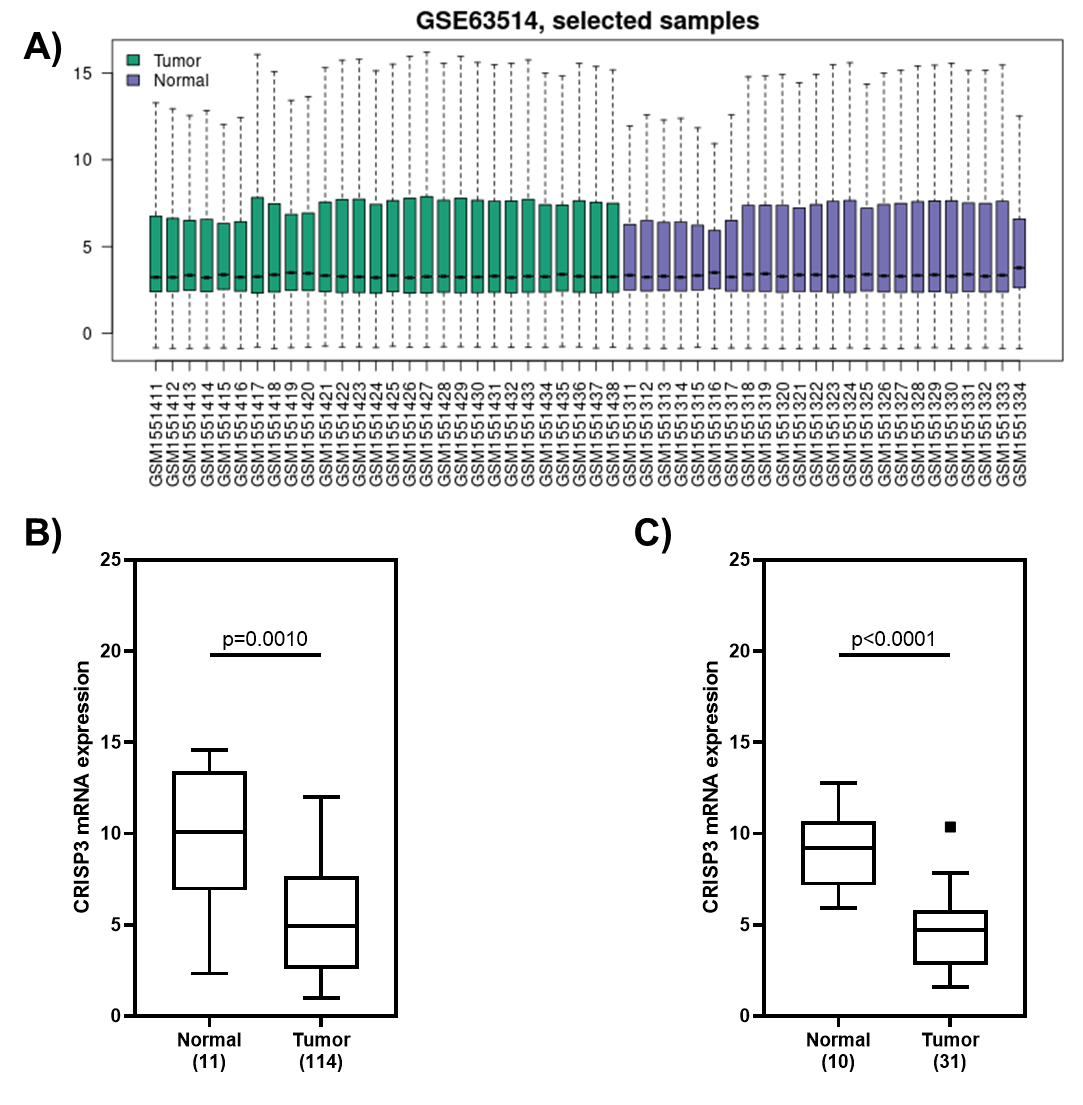


**Supplementary Figure 1.** Box plot of dataset samples standardization using GEO2R. Only normal (24) and tumor (28) samples were used for DEGs analysis. DEGs: differentially expressed genes. B) and C) CRISP3 mRNA expression in normal and tumor samples using the GPL570 platform (HG-U133_plus_2) and GPL96 platform (HG-U133A) in GENT2.


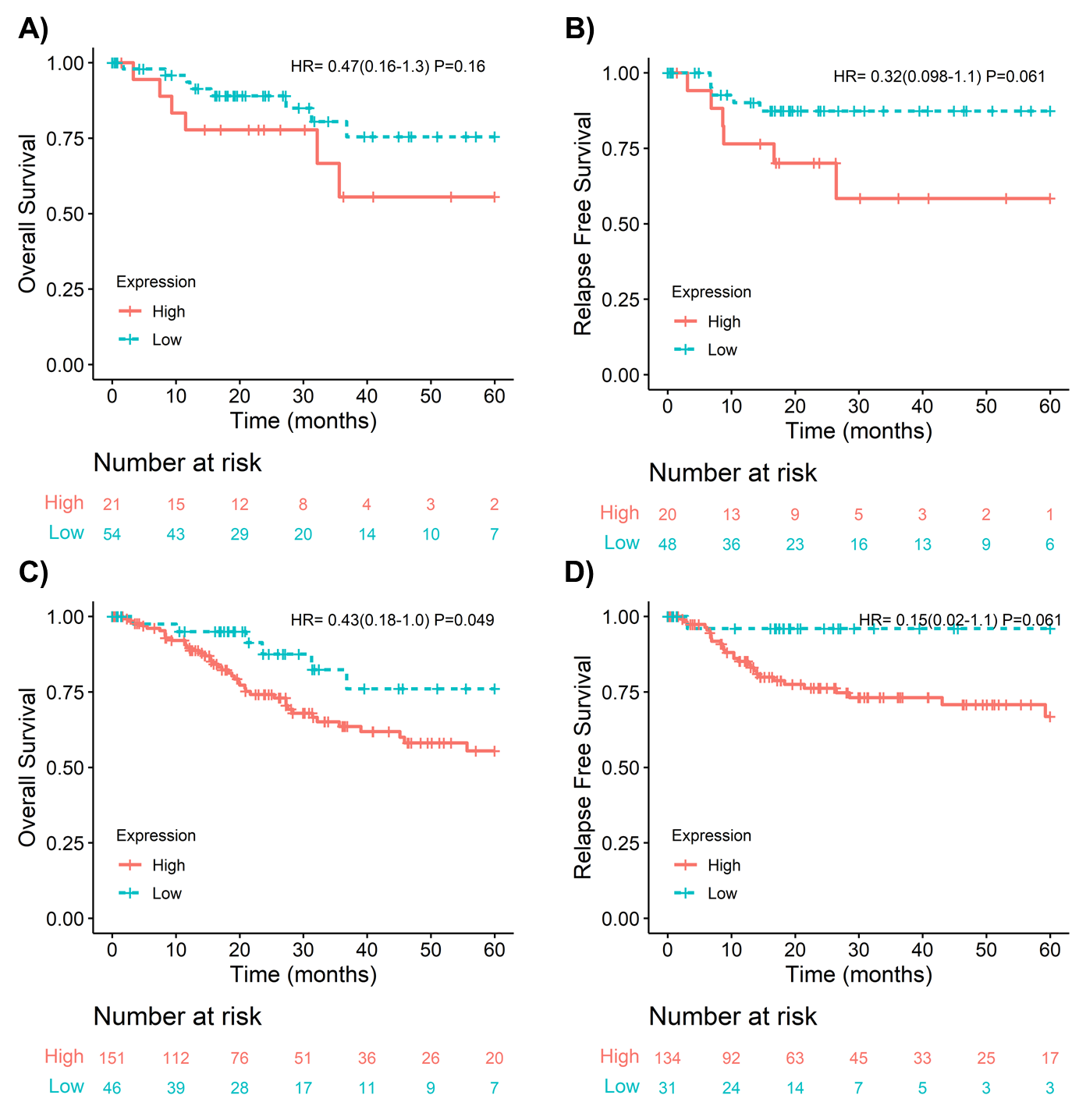


**Supplementary Figure 2.** Survival analysis of patients with squamous cell carcinomas of the uterine cervix according to hsa-miR-508-5p (A, B) and hsa-miR-3614-5p (C, D) expression. A, C) Overall survival and B, D) Relapse-free survival. Patients were categorized according to the best cutoff of microRNA expression. Data from the TCGA Firehose study.


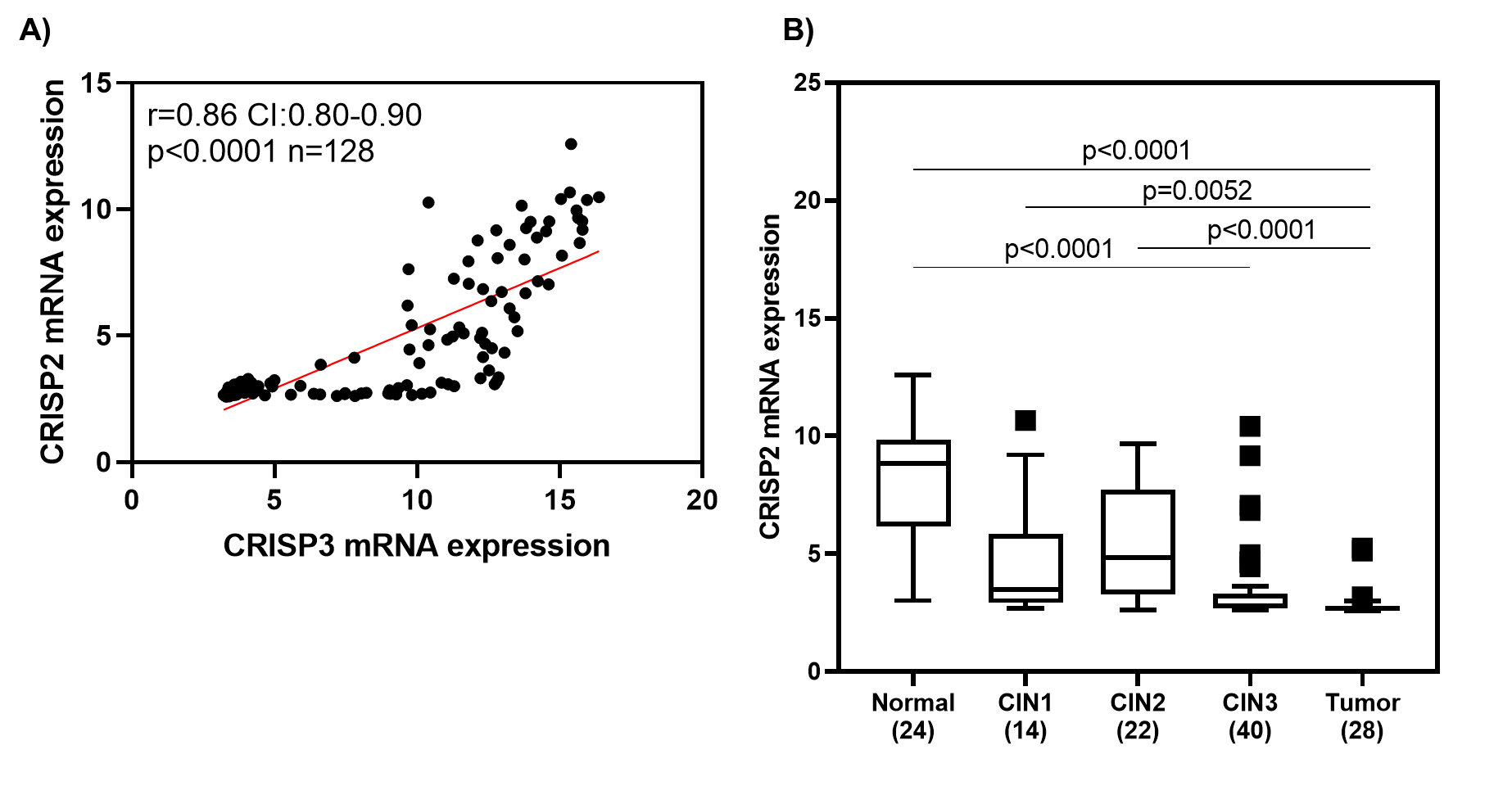
**Supplementary Figure 3.** A) Correlation between CRISP3 and CRISP2 RNA levels was accessed using the Spearman test. B) CRISP2 expression profile in normal tissue, grade 1, 2 and 3 of cervical intraepithelial neoplasia (CIN) and tumor tissue. The data were tested for normality and subjected to the Mann-Whitney and Kruskal-Wallis U Test with Dunn’s multiple comparison test. All the analyses are based on GSE63514 dataset.
